# Supplementary material for: Zinc-finger (ZiF) fold secreted effectors form a functionally diverse family across lineages of the blast fungus Magnaporthe oryzae
Source: PLoS Pathog. 2024 Jun 17;20(6):e1012277. doi: 10.1371/journal.ppat.1012277 (PMC11213319; doi:10.1371/journal.ppat.1012277)

Figure 1b – Uncropped blot images

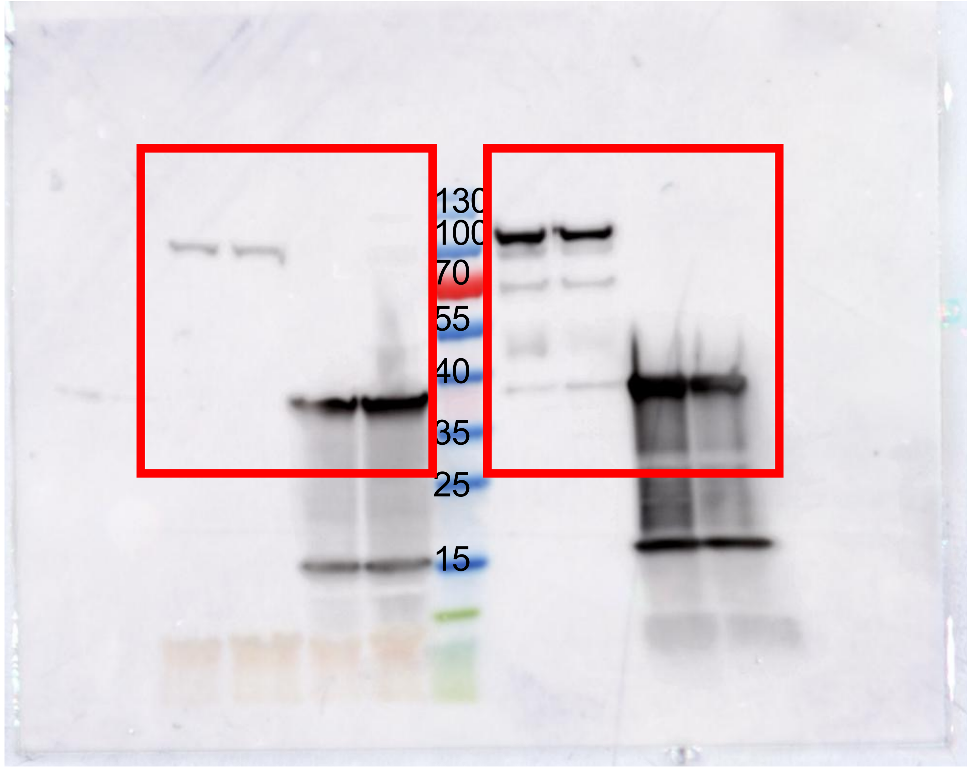

Anti-Flag membrane

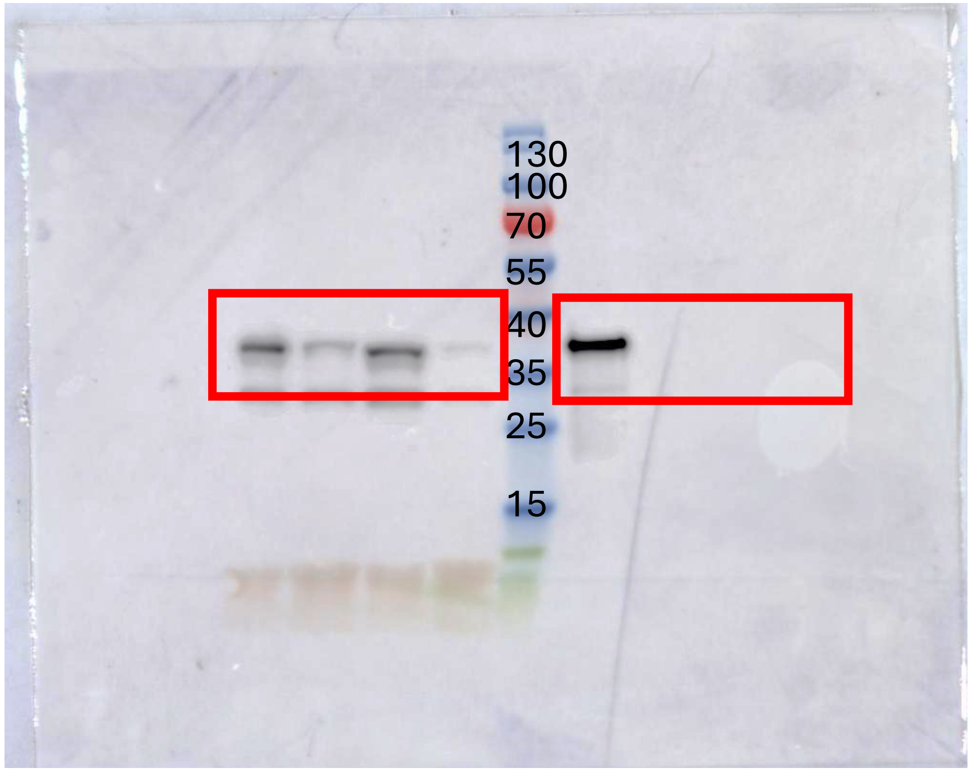

Anti-GFP membrane

Figure S1 – Uncropped blot images

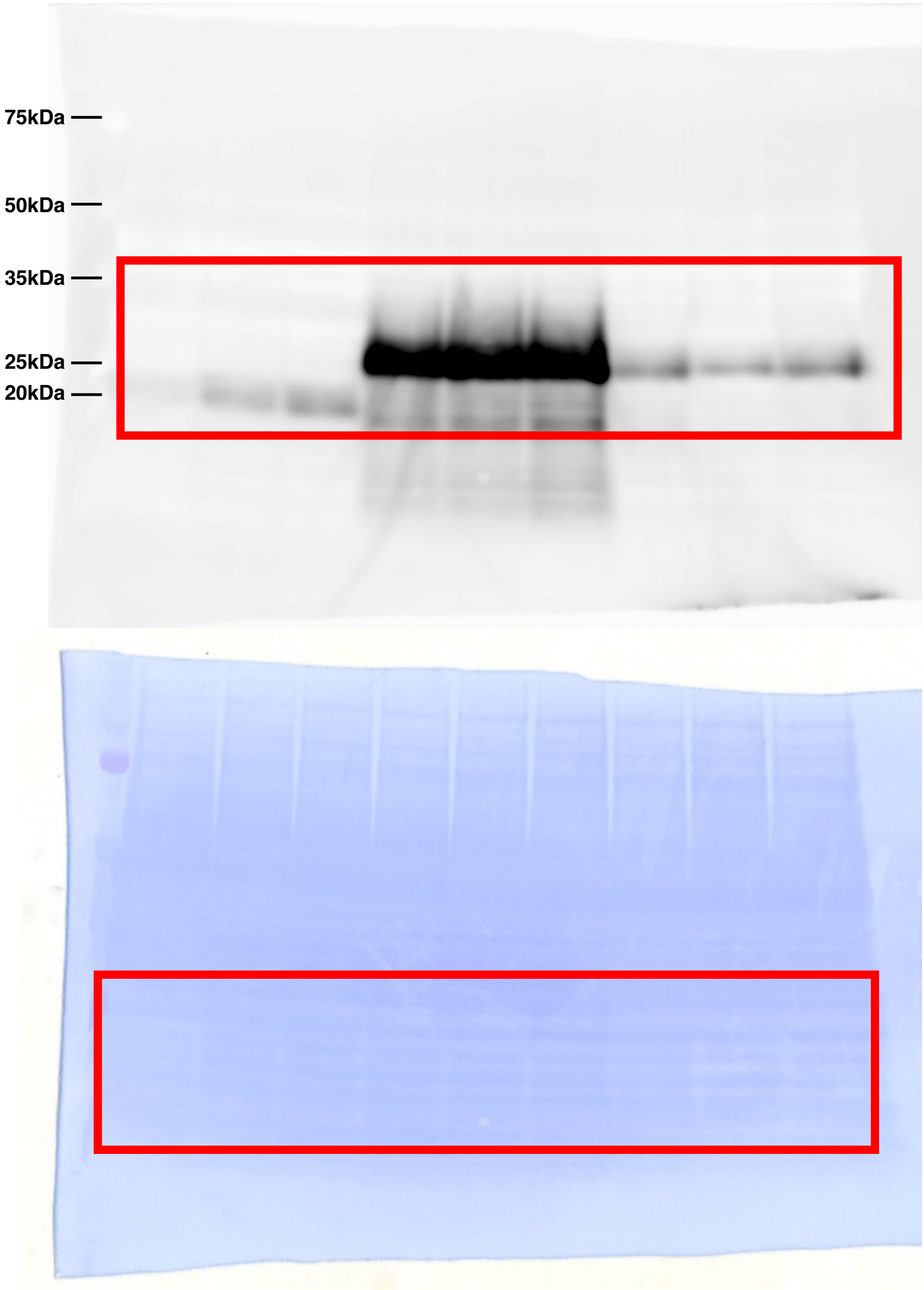

Figure S2a – Uncropped blot images

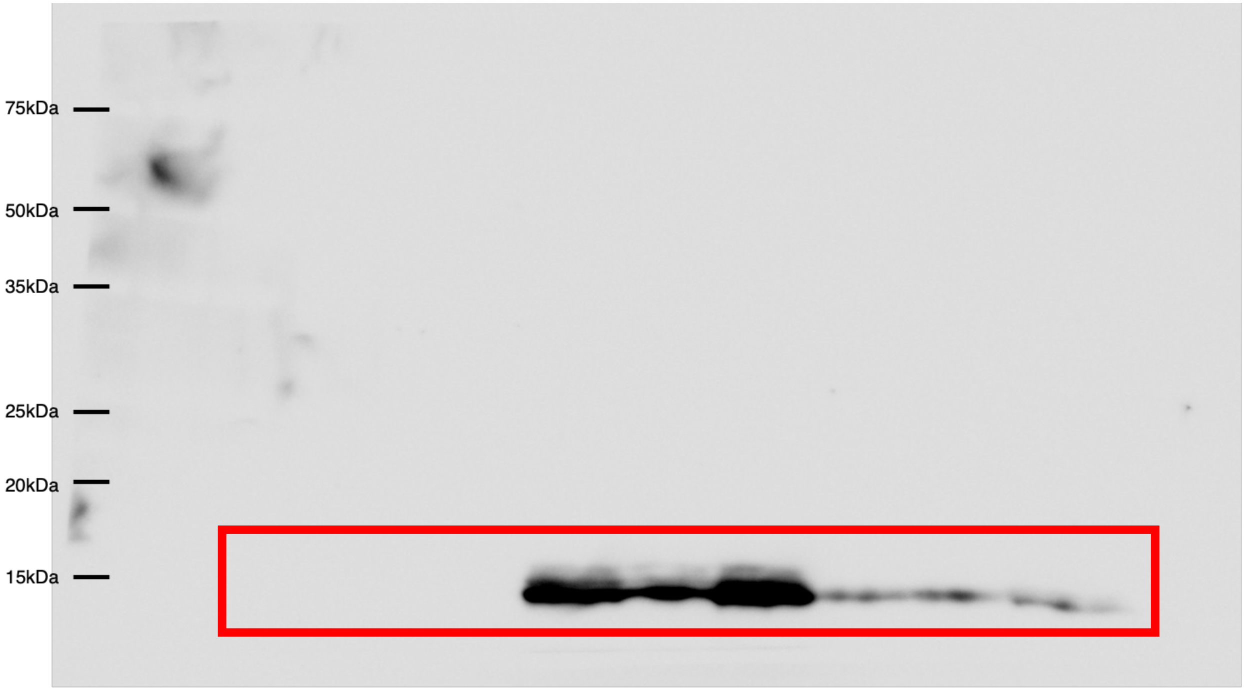

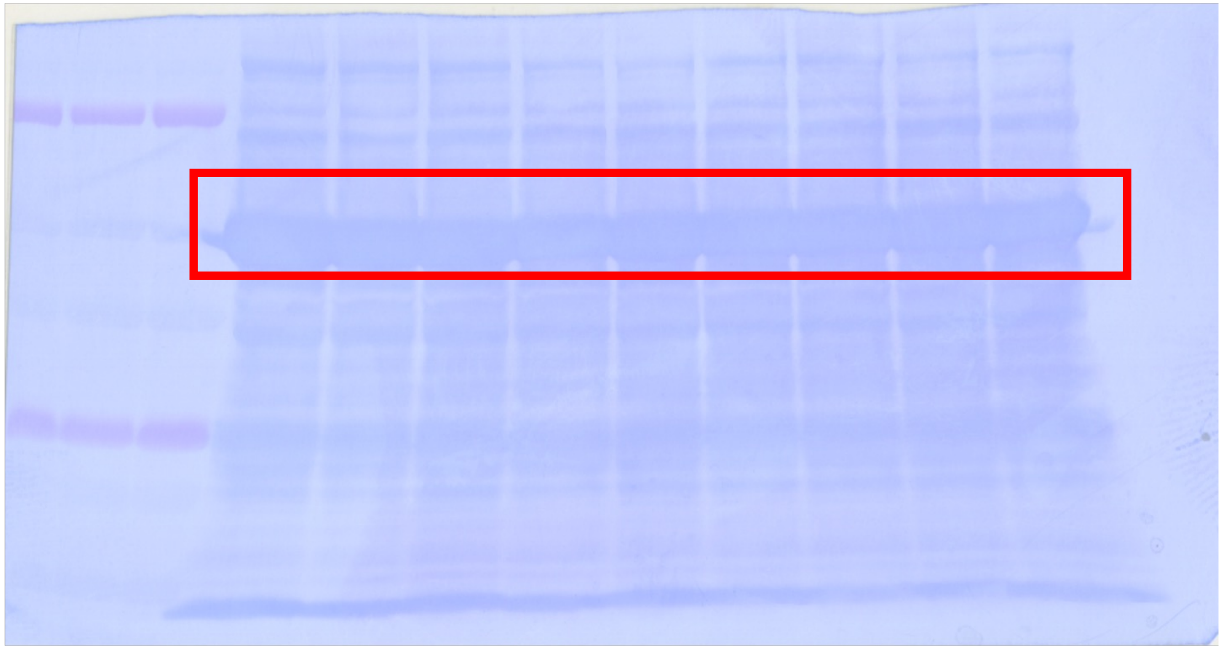

Figure S2b – Uncropped blot images

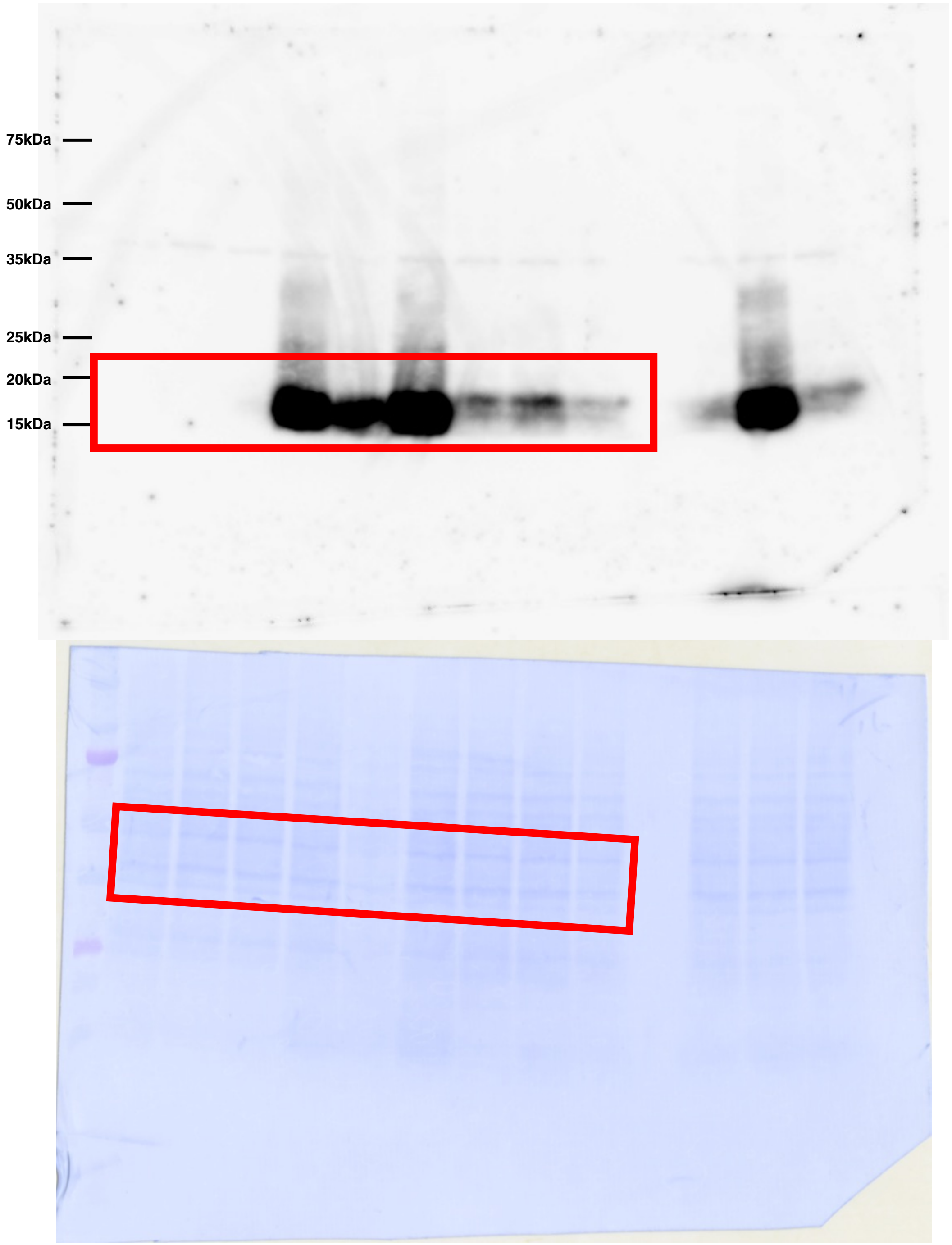

Figure S6 – Uncropped blot images

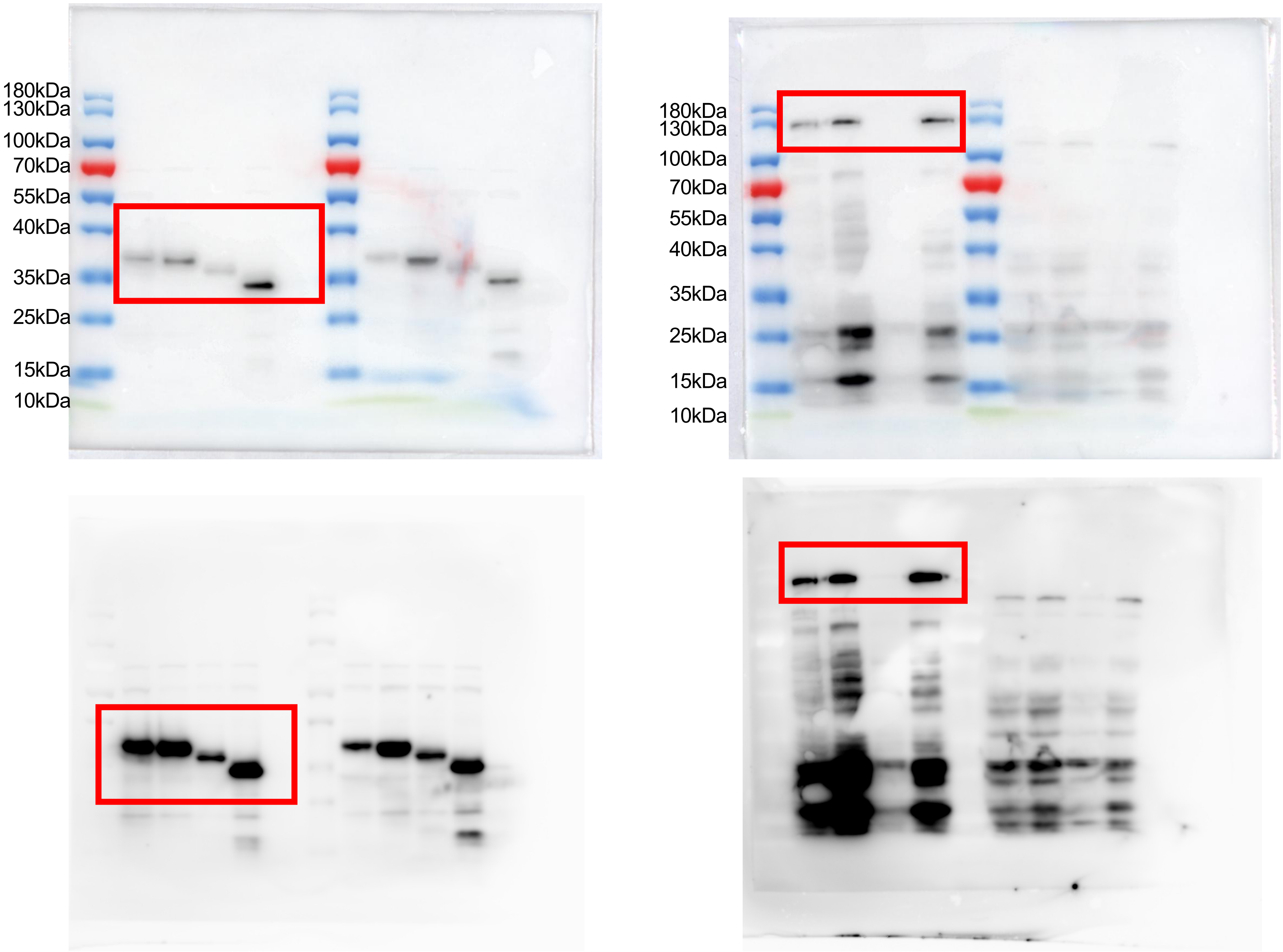

Figure S10 – Uncropped blot images

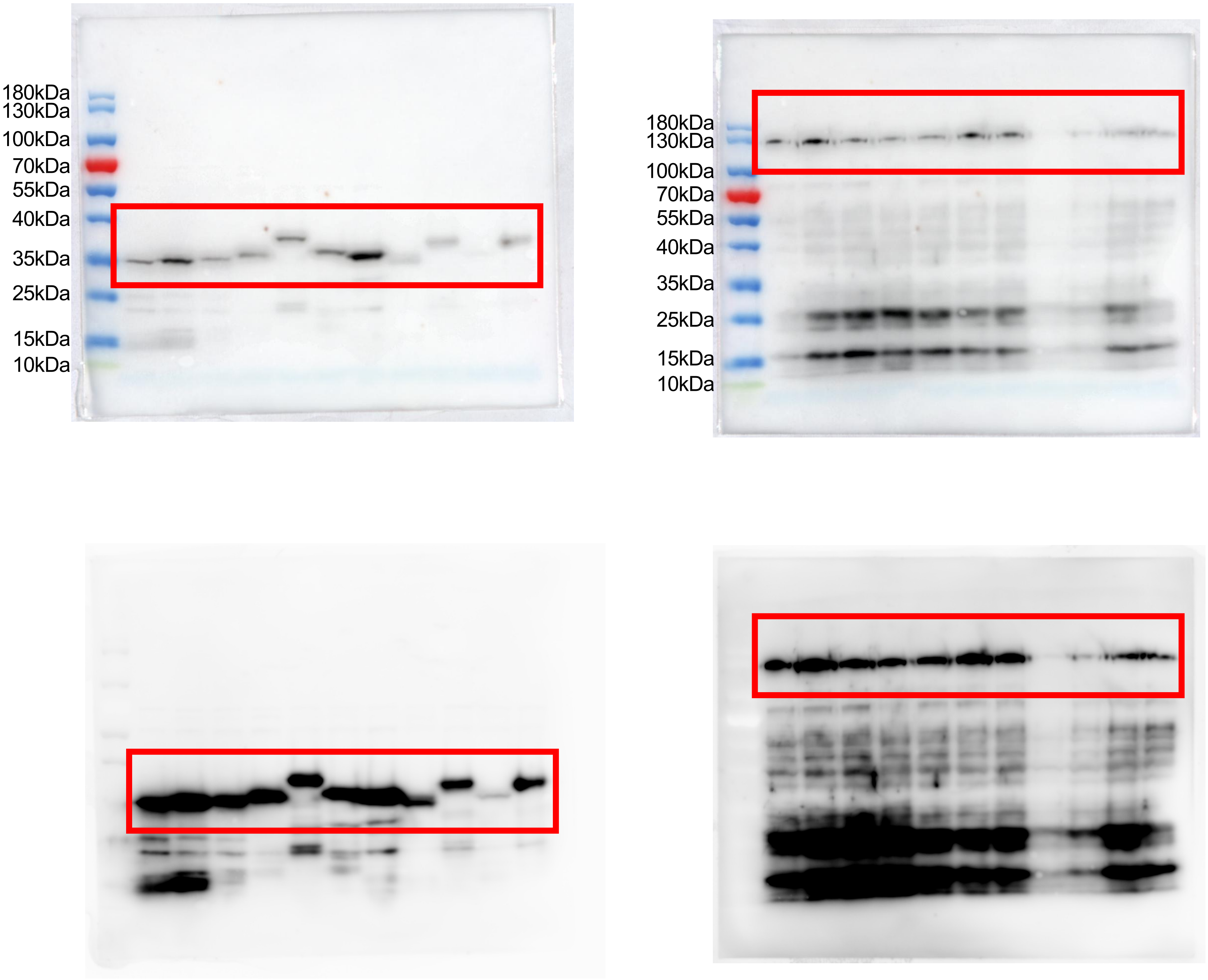

Figure S11 – Uncropped blot images

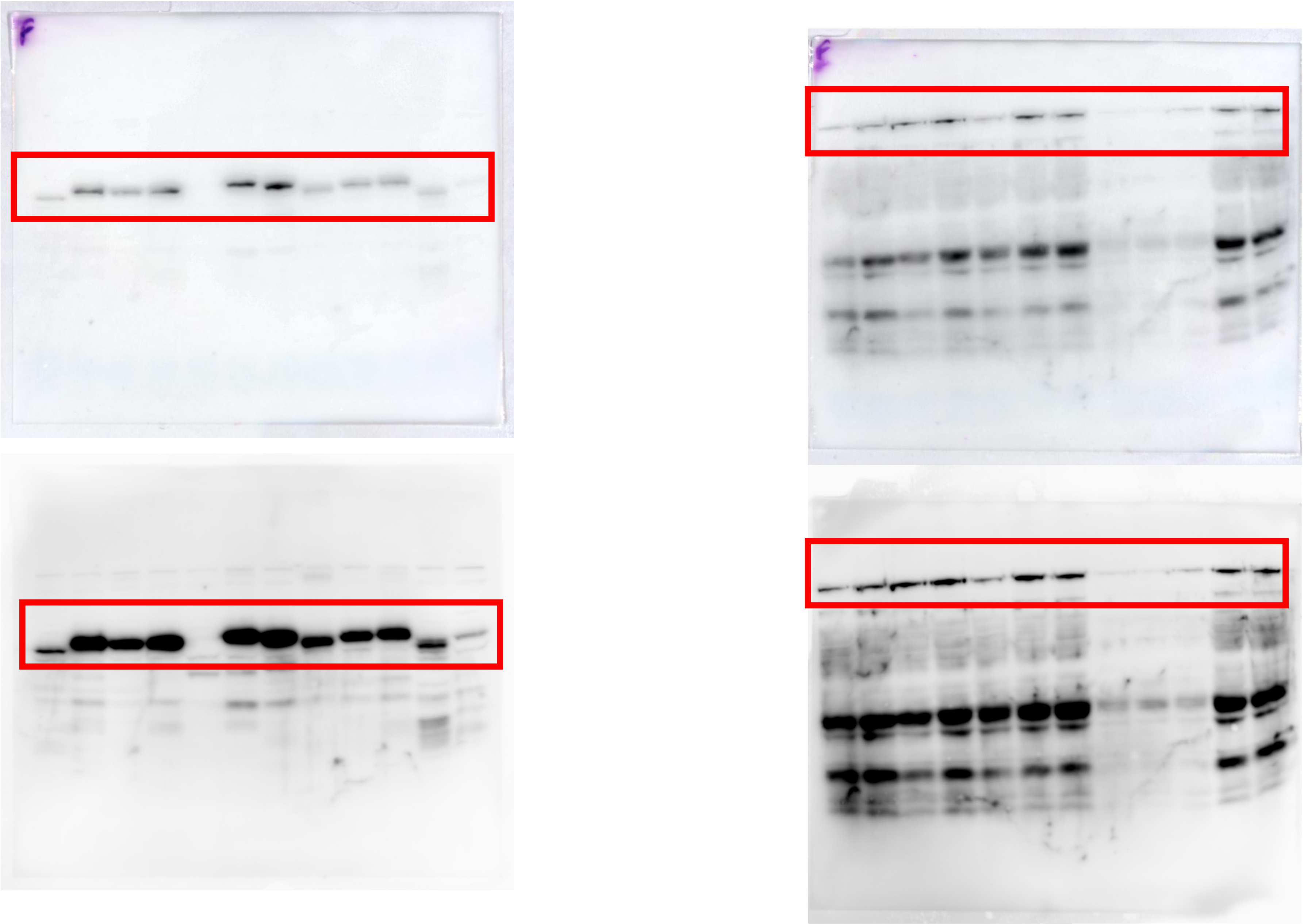

Supplement: S6 Data — (PDF) [file ppat.1012277.s006.pdf]
