# Supplementary figures and images for: Zinc-finger (ZiF) fold secreted effectors form a functionally diverse family across lineages of the blast fungus Magnaporthe oryzae
Source: PLoS Pathog. 2024 Jun 17;20(6):e1012277. doi: 10.1371/journal.ppat.1012277 (PMC11213319; doi:10.1371/journal.ppat.1012277)

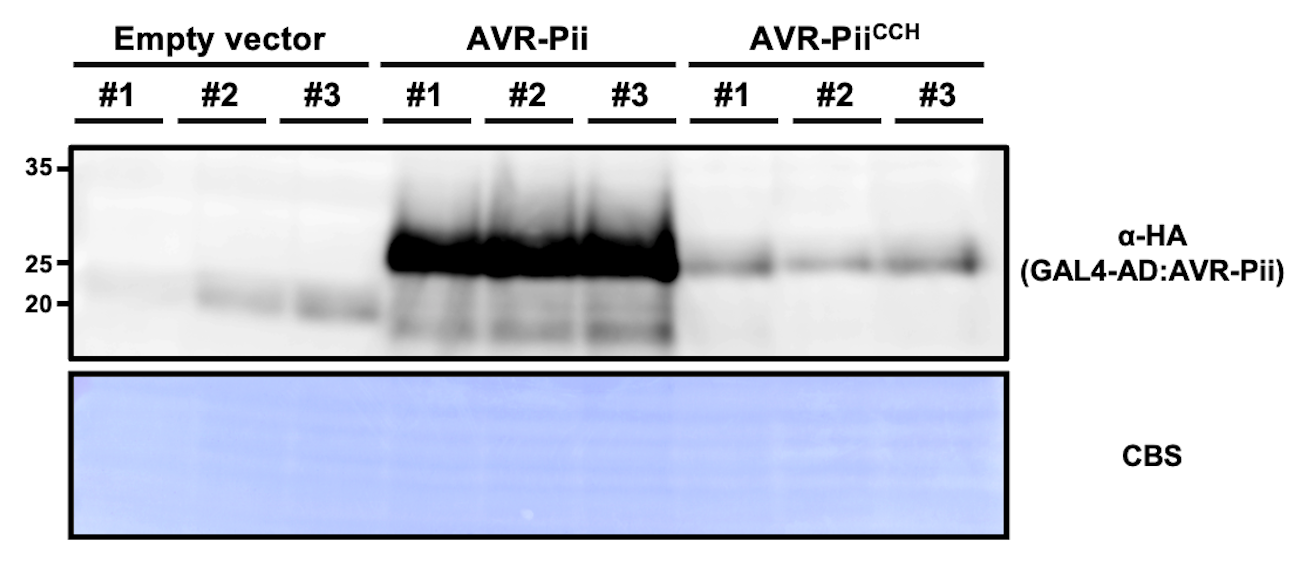

Supplement: S1 Fig — Yeast lysate was probed for the expression of AVR-Pii effectors using anti-HA antibodies for the effectors fused to the GAL4 activation domain (AD). Total protein extracts were coloured with Coomassie Blue Stain (CBS). (TIFF) [file ppat.1012277.s007.tiff]

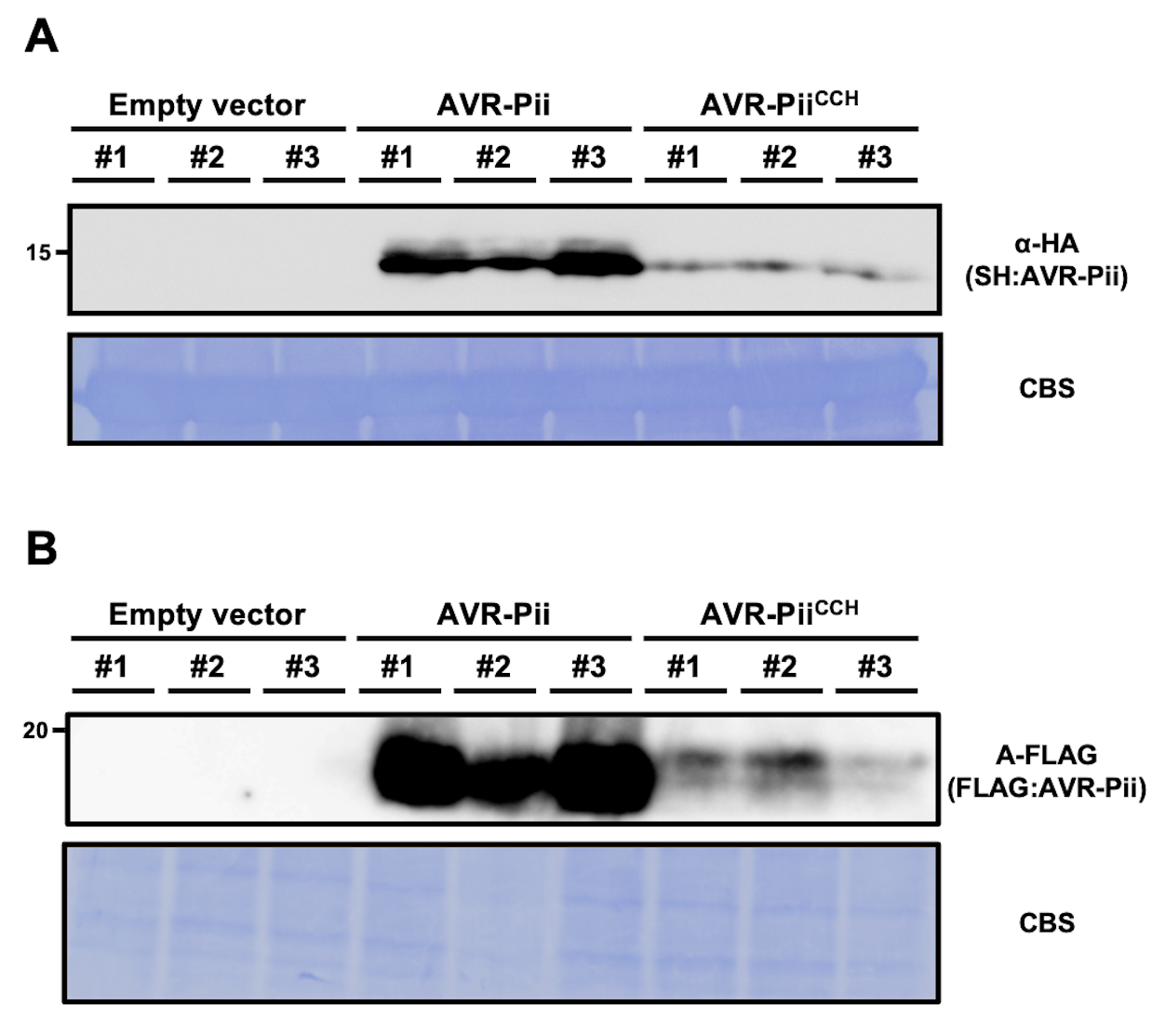

Supplement: S2 Fig — (A) Western blot analysis of N-terminally SH-tagged AVR-Pii or AVR-PiiCCH transiently expressed in N. benthamiana. Plant lysates were probed with anti-HA antibodies for the presence of effectors. Total protein extracts were colored with Coomassie Blue Stain (CBS). (B) Western blot analysis of N-terminally 3xFLAG-tagged AVR-Pii or AVR-PiiCCH transiently expressed in rice protoplasts. Lysates from rice protoplasts were probed with anti-FLAG antibodies for the presence of AVR-Pii effectors. Total protein extracts were colored with Coomassie Blue Stain (CBS). (TIFF) [file ppat.1012277.s008.tiff]

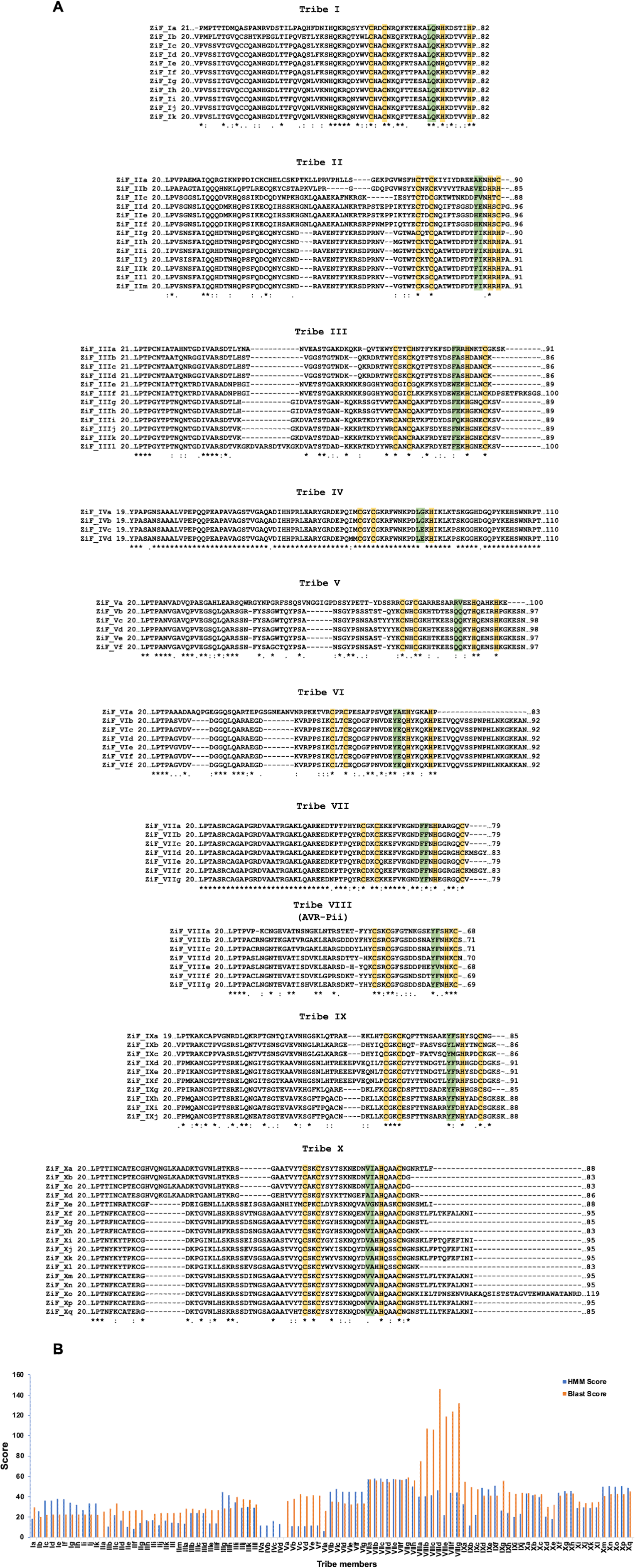

Supplement: S3 Fig — (A) Alignment of ZiF effector tribes showing conservation of the Zinc-finger motif and differences in residues forming the Exo70 binding interface. Protein sequence alignment of ZiF effector proteins reported here separated by tribes generated with Clustal Omega [71]. Residues contributing to the formation of a Zinc-finger motif are highlighted in yellow and residues at the equivalent positions of AVR-Pii binding interface [43] are highlighted in green. (B) Scores from hmmsearch and BLASTP search using ZiF HMM and Avr-Pii, respectively, against ZiF effector tribes. (TIFF) [file ppat.1012277.s009.tiff]

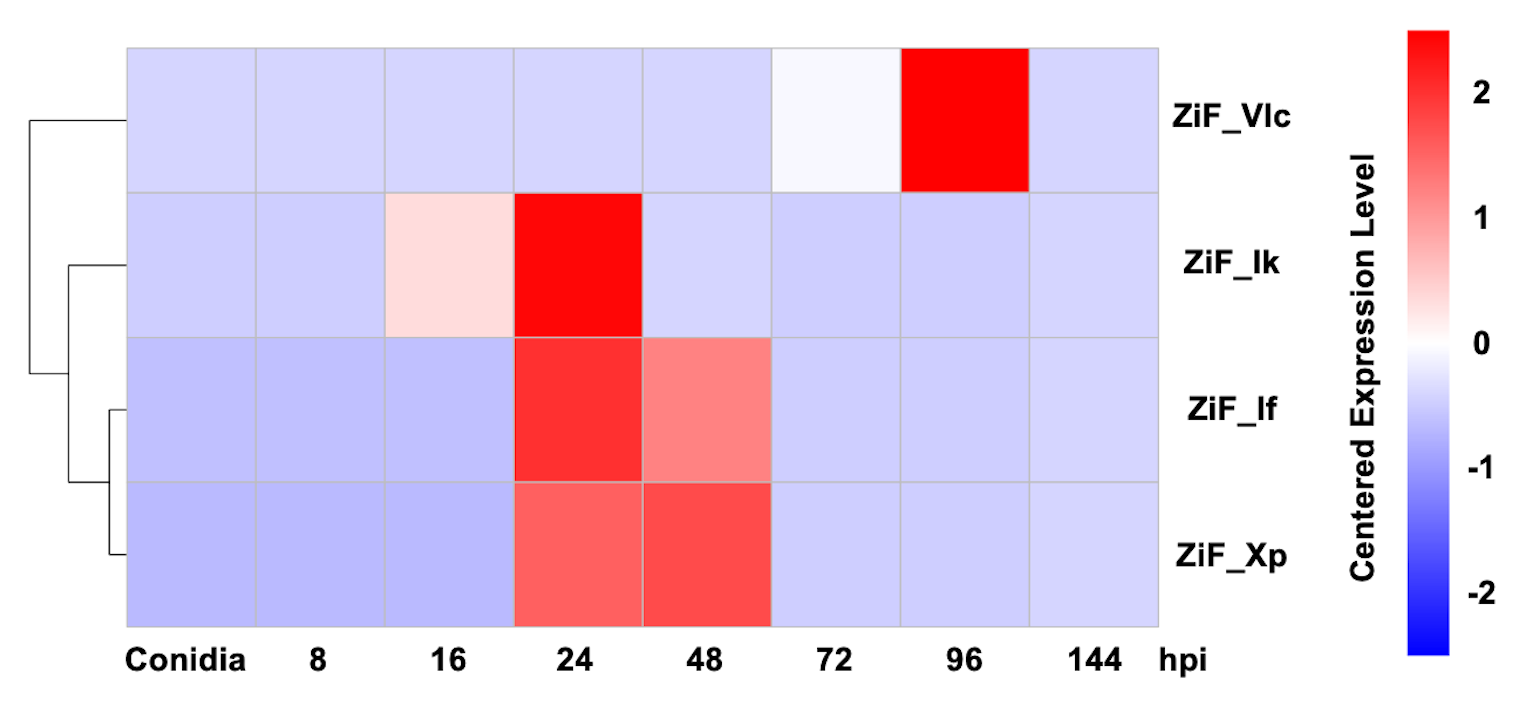

Supplement: S4 Fig — Heatmap for expression of ZiF effectors at different times after infection (hpi). Expression is represented in shades of red or blue according to the centered expression levels as indicated. Only effectors for which we found expression are represented. Magnaporthe isolate Guy11 does not harbour AVR-Pii (ZiF_VIII). (TIFF) [file ppat.1012277.s010.tiff]

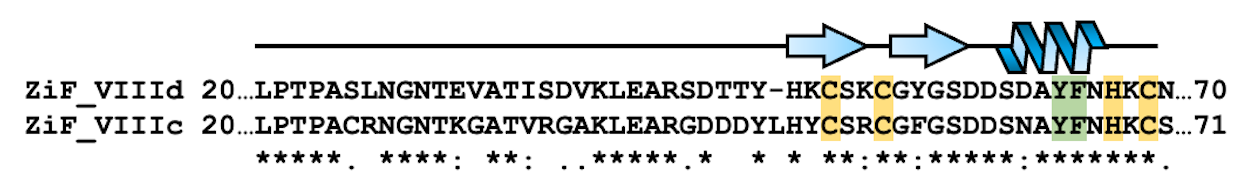

Supplement: S5 Fig — Protein sequence alignment of ZiF effector proteins ZiF_VIIId (AVR-Pii) and ZiF_VIIIc with Clustal Omega [71]. Secondary structure features based on AVR-Pii structure [43] are shown above. Residues contributing to the formation of a Zinc-finger motif are highlighted in yellow whereas the residues at the equivalent positions of AVR-Pii binding interface [43] are highlighted in green. (TIFF) [file ppat.1012277.s011.tiff]

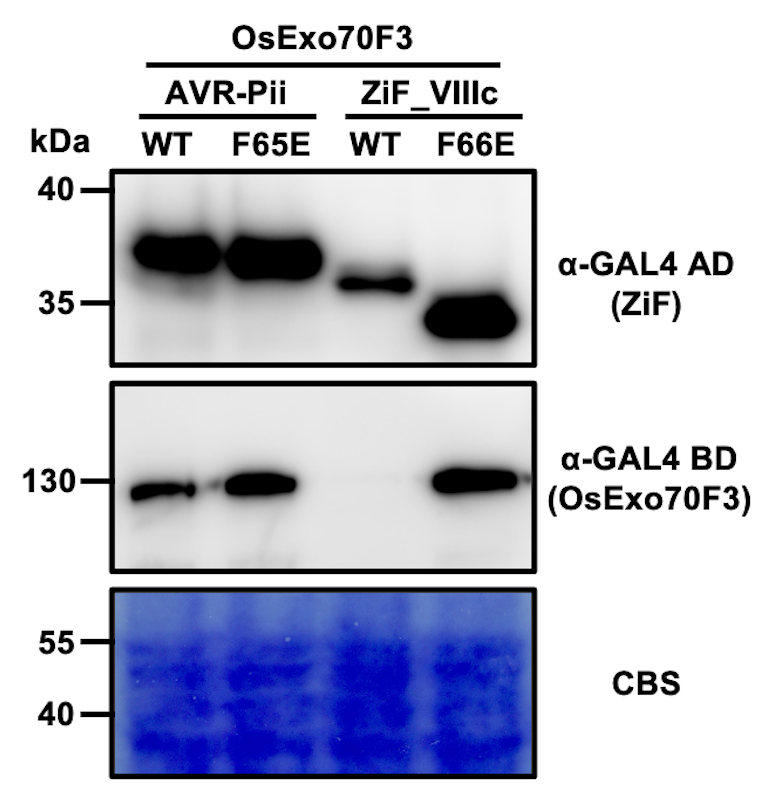

Supplement: S6 Fig — Yeast lysate was probed for the presence of OsExo70F3, AVR-Pii, ZiF_VIIIc and their respective mutants using anti-GAL4 binding domain (BD) and anti-GAL4 DNA activation domain (AD) antibodies. Total protein extracts were stained with Coomassie Blue Stain (CBS). OsExo70F3 accumulation is consistently lower in positive interactions with ZiF effectors as noticed here and elsewhere in this and previous studies [43]. (TIFF) [file ppat.1012277.s012.tiff]

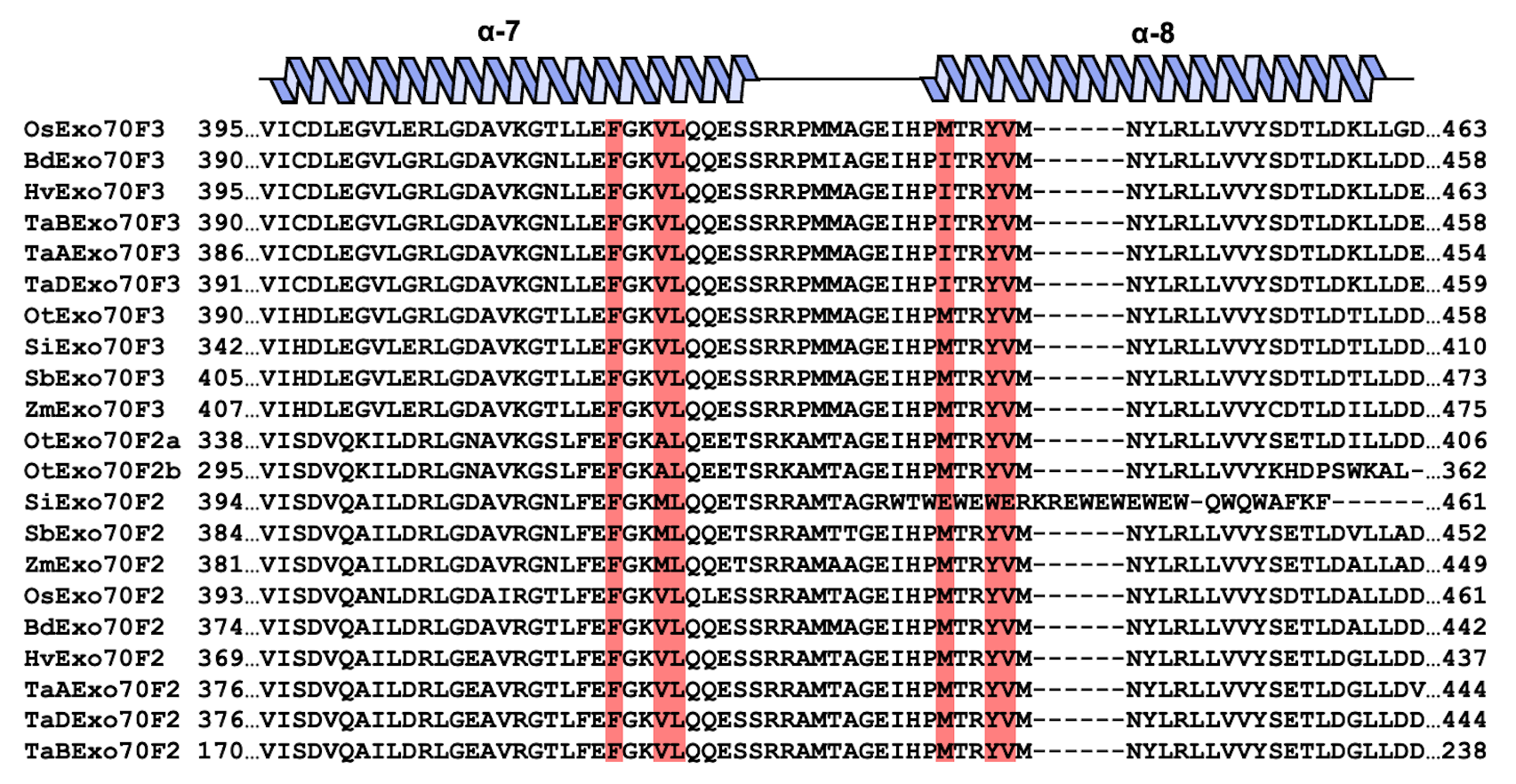

Supplement: S7 Fig — Protein sequence alignment for the orthologs of AVR-Pii host targets, OsExo70F2 and OsExo70F3, from different grass species annotated by Holden et al. [54] (Os: Oryza sativa; Bd: Brachypodium distachyon; Hv: Hordeum vulgare; Ta: Triticum aestivum; Ot: Oropetium thomaeum; Si: Setaria italica; Sb: Sorghum bicolor; Zm: Zea mays). Sequence alignment was generated with Clustal Omega [71]. Residues at the equivalent positions of the OsExo70F2 binding interface with AVR-Pii are highlighted in red. Secondary structure features based on OsExo70F2 structure [43] are shown above. (TIFF) [file ppat.1012277.s013.tiff]

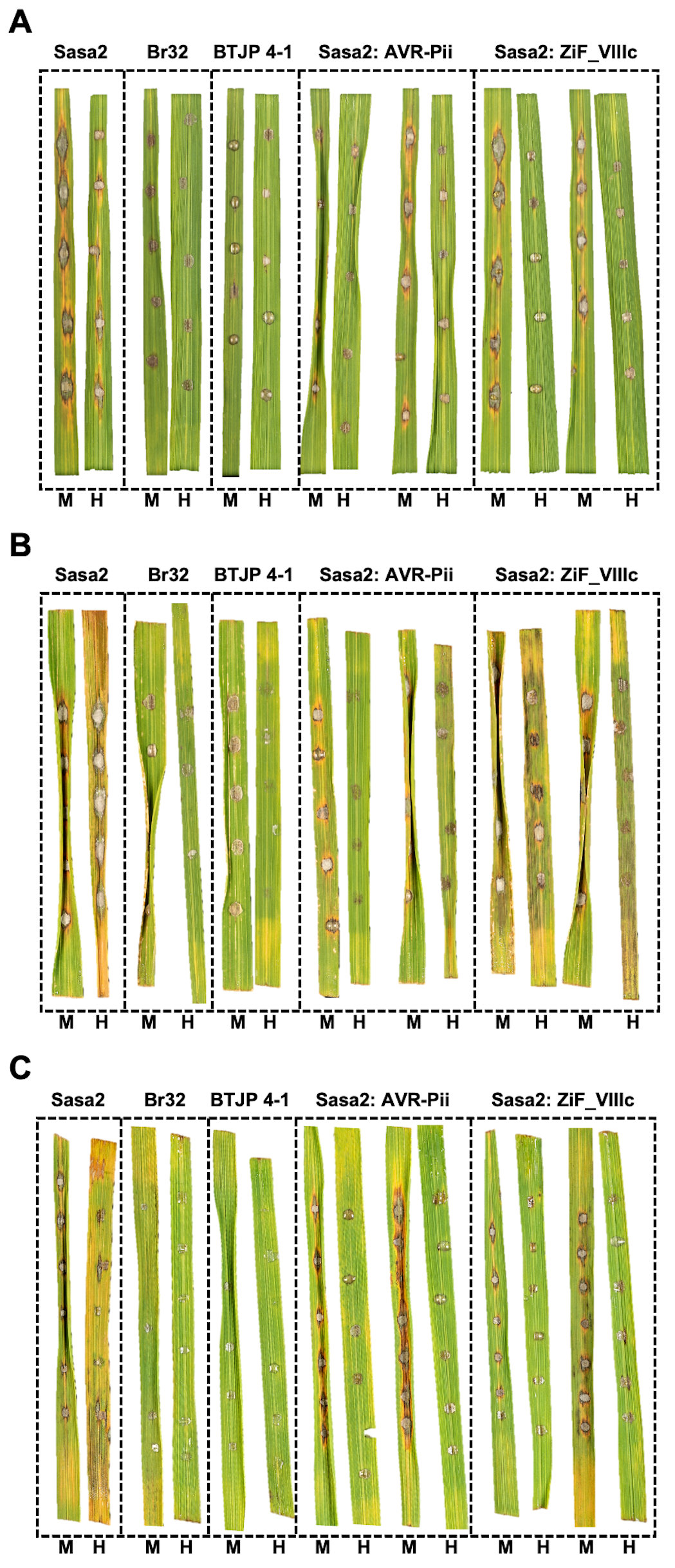

Supplement: S8 Fig — First (A), second (B) and third (C) replicate of the rice leaf blade spot inoculation assay presented in Fig 4. Transgenic M. oryzae Sasa2 harbouring AVR-Pii or ZiF_VIIIc were spotted into rice cultivars Moukoto (Pii-) and Hitomebore (Pii+). The cultivars Moukoto and Hitomebore are denoted by M and H, respectively. Wild-type rice blast isolate Sasa2 and wheat blast Br32 and BTJP 4–1 are included as controls. (TIFF) [file ppat.1012277.s014.tiff]

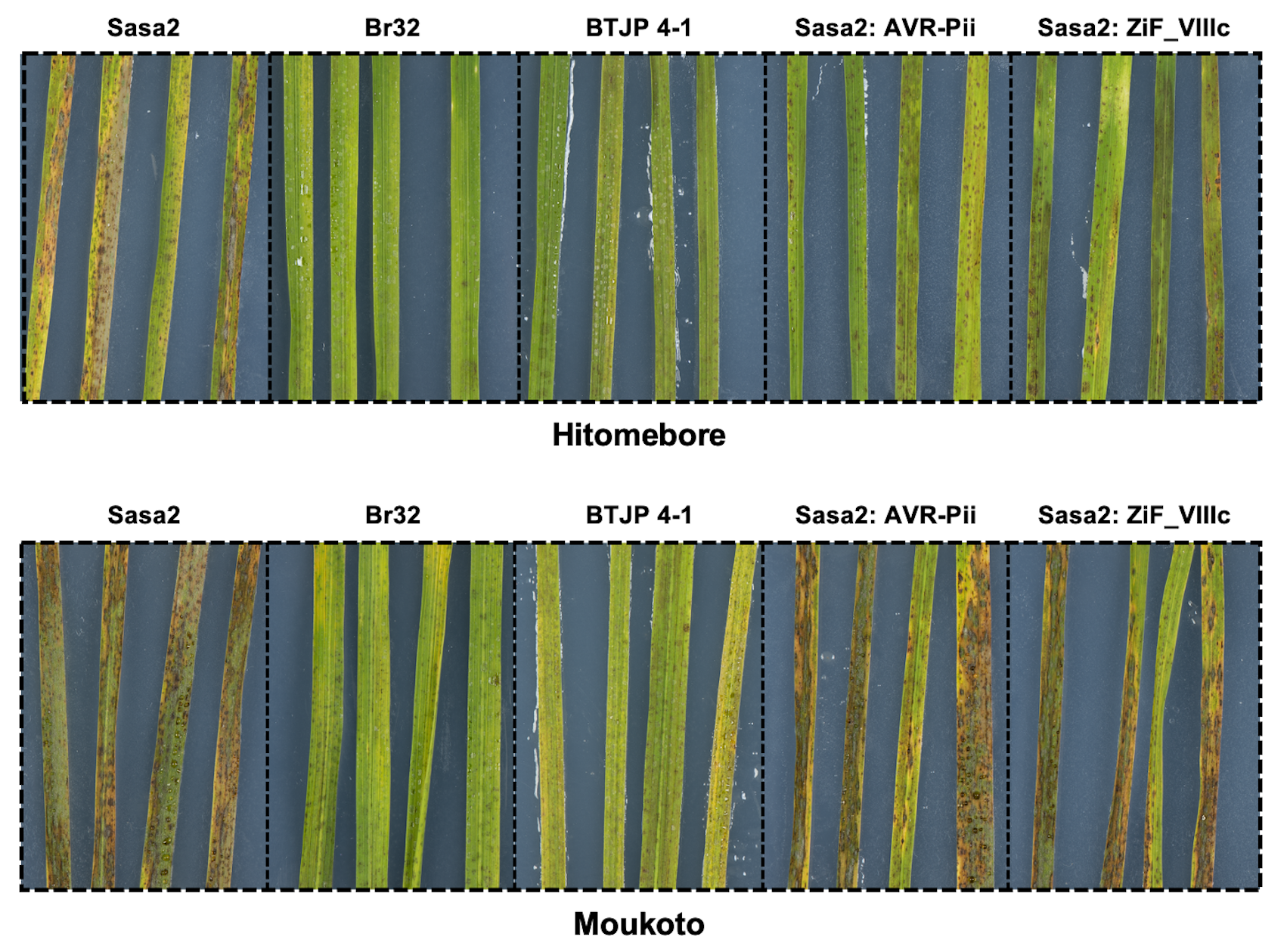

Supplement: S9 Fig — Four replicates of the rice leaf blade spray inoculation assay presented in Fig 4. Conidia from transgenic isolates of M. oryzae Sasa2 harbouring AVR-Pii or ZiF_VIIIc were sprayed into rice cultivars Moukoto (Pii-) and Hitomebore (Pii+) at a concentration of 1X105 conidia mL-1 in 0.2% gelatine. Images were taken at 5 days post-infection. Wild-type rice blast isolate Sasa2 and wheat blast Br32 and BTJP 4–1 are included as controls. (TIFF) [file ppat.1012277.s015.tiff]

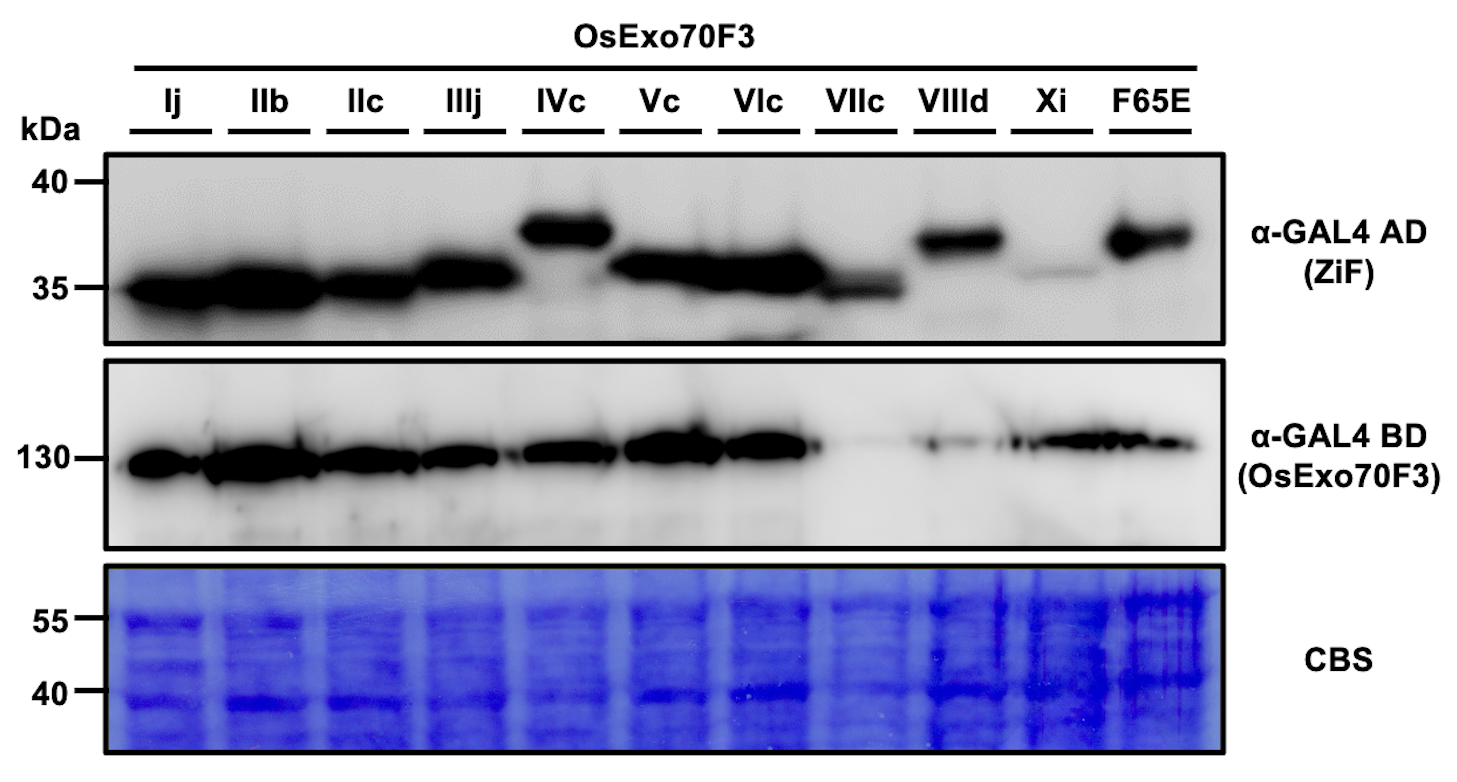

Supplement: S10 Fig — Yeast lysate was probed for the presence of OsExo70F3 using anti-GAL4 binding domain (BD) and the accumulation of selected rice blast ZiF effectors was probed with anti-GAL4 DNA activation domain (AD) antibodies. Total protein extracts were stained with Coomassie Blue Stain (CBS). OsExo70F3 accumulation is consistently lower in positive interactions with ZiF effectors as noticed here and elsewhere in this and previous studies [43]. (TIFF) [file ppat.1012277.s016.tiff]

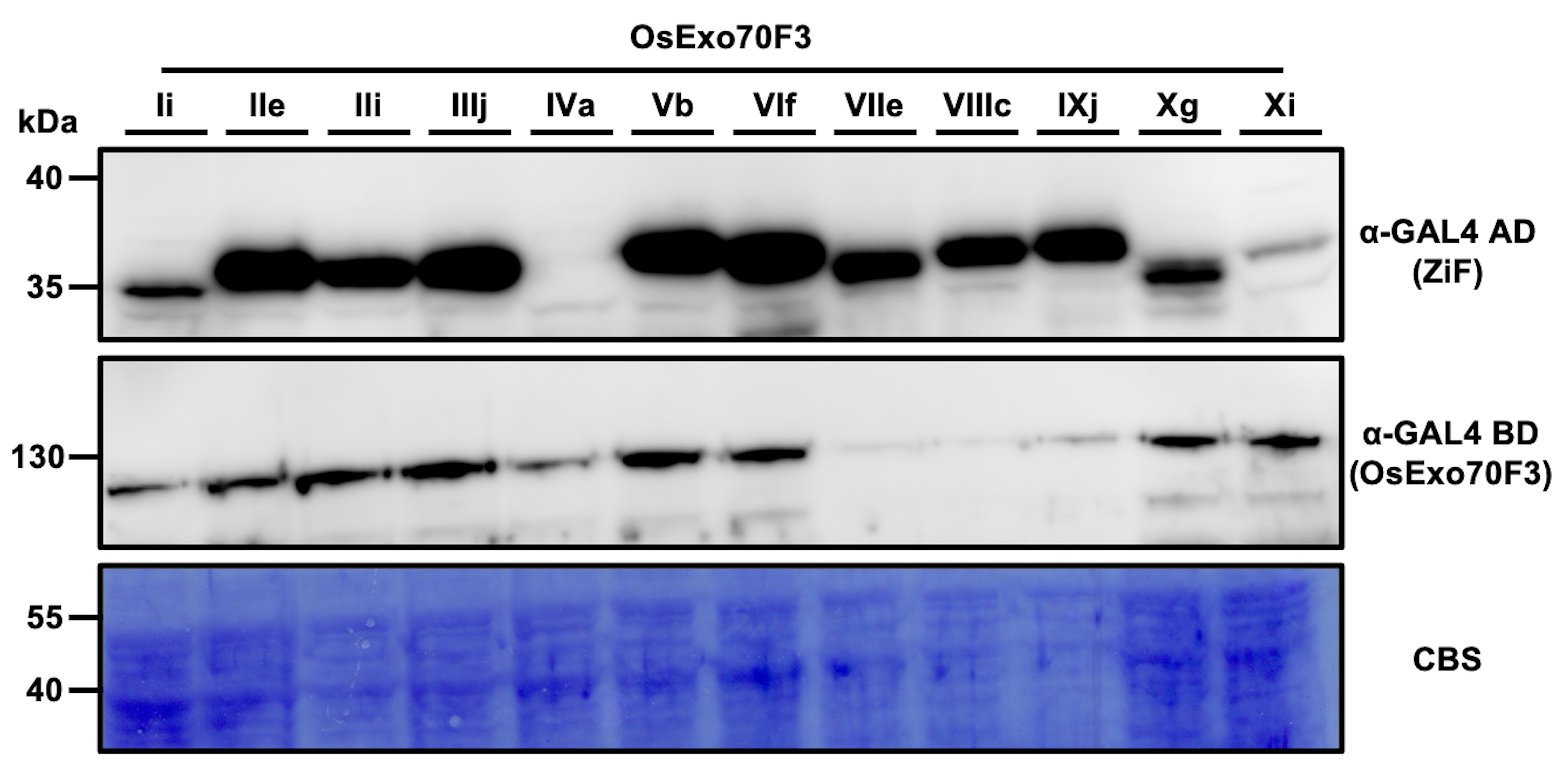

Supplement: S11 Fig — Yeast lysate was probed for the presence of OsExo70F3 using anti-GAL4 binding domain (BD) while the accumulation of selected ZiF effectors from wheat blast lineages was probed with anti-GAL4 DNA activation domain (AD) antibodies. For technical feasibility, positive and negative controls AVR-Pii and AVR-Pii Phe65Glu were not included in the western blot as their production in yeast cells was tested before [43] and elsewhere in this study. Total protein extracts were stained with Coomassie Blue Stain (CBS). OsExo70F3 accumulation is consistently lower in positive interactions with ZiF effectors as noticed here and elsewhere in this and previous studies [43]. Due to the number of samples loaded on the SDS-PAGE gel before blotting, the molecular mass markers used in this specific figure are based on those of S10 Fig (see S6 Data for all uncropped blots). (TIFF) [file ppat.1012277.s017.tiff]
